# Supplementary material for: Influence of glucose on swarming and quorum sensing of Dickeya solani
Source: PLoS One. 2022 Feb 22;17(2):e0263124. doi: 10.1371/journal.pone.0263124 (PMC8863224; doi:10.1371/journal.pone.0263124)
Supplement: Attachment — Submitted filename: review.docx [file pone.0263124.s001.docx]

Please clearly explain the aims of the results and study…

there were some grammar or writing mistakes..

33 signaling molecules are different depending on the volume of the medium in the plate.
34 Author summary
 In this study, we show that the swarming motility of Dickeya
41 solani and production of the quorum-sensing molecules are coupled with the central ….
44 Introduction
64
65 towards their source, is most probably ….
92 for 24 and 48 has indicated.

The 48-hour incubation enabled the detection of AHL

204 bacteria can penetrate them through wounds and natural openings,
219 response to such conditions, bacteria could trigger swarming motility to enable optimally
232 swarming but not swimming motility of *Pseudomonas aeruginosa*, is
233dependent on changing concentrations of glucose in the medium [32]. um
271 sensing signaling molecules in the medium prevent the triggering of swarming.
Materials and Methods
278 Bacterial strains and media
279 Dickeya solani strain IFB102 (21) was used as a wild-type strain.

Best regard,

S. ABBASI
