# Supplementary material for: Influence of glucose on swarming and quorum sensing of Dickeya solani
Source: PLoS One. 2022 Feb 22;17(2):e0263124. doi: 10.1371/journal.pone.0263124 (PMC8863224; doi:10.1371/journal.pone.0263124)
Supplement: Attachment — Submitted filename: Response to Reviewers.docx [file pone.0263124.s003.docx]

PLOS ONE

Editorial Board

Gdańsk, 09 August 2021

Dear Dr. Manjunath Hubballi,

I am sending a revised version of the manuscript [PONE-D-21-19894] along with responses to reviewers' comments.

Specific answers to reviewers:

Reviewer #1:

The aims of the study have been clearly explained in the abstract section (lines 28-31).

The manuscript has been corrected according to Reviewer’s suggestions (lines: 33, 42, 66, 102, 134, 162, 170, 205, 220-221,272, 280).

Reviewer #2:

To our knowledge, this is the first study performed on *Dickeya solani* in which swarming motility of this bacterium was shown to depend on the volume on the medium in the cultivation plate and glucose content. While the influence of the analyzed sugar on the swarming motility was shown by other authors in context of other bacterial species there are no studies documenting such phenomenon in the case of *D. solani*.

Reviewer #3:

We thank very much for kind appreciation of our work.

My best regards,

Michał Obuchowski (corresponding author)

Division of Molecular Bacteriology

Intercollegiate Faculty of Biotechnology UG & MUG

Medical University of Gdańsk

Skłodowskiej-Curie 3a

80-210 Gdańsk

Poland
